# Evolution and genome specialization of *Brucella suis* biovar 2 Iberian lineages

**DOI:** 10.1186/s12864-017-4113-8

**Published:** 2017-09-12

**Authors:** Ana Cristina Ferreira, Rogério Tenreiro, Maria Inácia Corrêa de Sá, Ricardo Dias

**Affiliations:** 1 Instituto Nacional de Investigação Agrária e Veterinária, I.P. (INIAV, IP), Av. da República, Quinta do Marquês, 2780-157 Oeiras, Portugal; 2 Universidade de Lisboa, Faculdade de Ciências, Biosystems and Integrative Sciences Institute (BioISI), Edificio TecLabs, Campus da FCUL, Campo Grande, 1749-016 Lisbon, Portugal

**Keywords:** *Brucella* spp., *B. suis* biovar 2, Phylogenomics, comparative genomics, Iberian ecovar

## Abstract

**Background:**

Swine brucellosis caused by *B. suis* biovar 2 is an emergent disease in domestic pigs in Europe. The emergence of this pathogen has been linked to the increase of extensive pig farms and the high density of infected wild boars (*Sus scrofa*). In Portugal and Spain, the majority of strains share specific molecular characteristics, which allowed establishing an Iberian clonal lineage. However, several strains isolated from wild boars in the North-East region of Spain are similar to strains isolated in different Central European countries.

**Results:**

Comparative analysis of five newly fully sequenced *B. suis* biovar 2 strains belonging to the main circulating clones in Iberian Peninsula, with publicly available *Brucella* spp. genomes, revealed that strains from Iberian clonal lineage share 74% similarity with those reference genomes. Besides the 210 kb translocation event present in all biovar 2 strains, an inversion with 944 kb was presented in chromosome I of strains from the Iberian clone. At left and right crossover points, the inversion disrupted a TRAP dicarboxylate transporter, DctM subunit, and an integral membrane protein TerC. The gene *dctM* is well conserved in *Brucella* spp. except in strains from the Iberian clonal lineage. Intraspecies comparative analysis also exposed a number of biovar-, haplotype- and strain-specific insertion-deletion (INDELs) events and single nucleotide polymorphisms (SNPs) that could explain differences in virulence and host specificities. Most discriminative mutations were associated to membrane related molecules (29%) and enzymes involved in catabolism processes (20%). Molecular identification of both *B. suis* biovar 2 clonal lineages could be easily achieved using the target-PCR procedures established in this work for the evaluated INDELs.

**Conclusion:**

Whole-genome analyses supports that the *B. suis* biovar 2 Iberian clonal lineage evolved from the Central-European lineage and suggests that the genomic specialization of this pathogen in the Iberian Peninsula is independent of a specific genomic event(s), but instead driven by allopatric speciation, resulting in the establishment of a new ecovar.

**Electronic supplementary material:**

The online version of this article (10.1186/s12864-017-4113-8) contains supplementary material, which is available to authorized users.

## Background

*Brucella suis* is a facultative intracellular pathogen infecting a broad range of animals and humans. This species comprises five biovars, denominated biovar 1 to 5, which infect specific hosts. Different molecular approaches have shown a considerable diversification within the *B. suis* clade and probable relationships among the biovars and their preferential hosts were suggested [1–4]. Biovars 1, 2 and 3 infect suidae and are the etiological agents of swine brucellosis, but biovars 1 and 3 differ in host specificity and geographical distribution from biovar 2. In the public health context, biovar 2 is rarely pathogenic, whereas biovars 1 and 3 are highly pathogenic and cause severe disease in humans [5]. *B. suis* biovar 2 was only described in Europe and also infects hares. It is generally accepted, that wild boars (*Sus scrofa*) and European hares (*Lepus europaeus*) are both wildlife reservoirs of this biovar and the source of transmission to backyard and extensively reared pigs [6]. Previous studies on the genetic structure of the *B. suis* biovar 2 population by Multiple Locus Variable-number Tandem Repeat Analysis (MLVA) [4, 7–10], PCR-RFLP analysis of *omp31*, *omp2a* and *omp2b* genes [10–13] and whole-genome optical mapping [11] supports the existence of two circulating lineages in the Iberian Peninsula (Portugal and Spain): a Central-European and a Iberian clonal lineage. The latter has been described exclusively in the Iberian Peninsula, in pigs and wild boars, mainly south of the River Ebro, suggesting a genomic specialization and local adaptation [12].

Pathogen fitness may vary from host to host. The way that pathogens evolve, within or outside hosts, and the strategy used by the host to resist infection can be determinant for pathogen specialization and an important driver of virulence evolution. Host specificity is associated with a number of genomic signatures, including genomic degeneration and genomic rearrangements [14]. Recently, the complete genome sequences of five *B. suis* biovar 2 strains representative of Iberian and Central-European clonal lineages, isolated from wild boars in Portugal and Spain, have been released [15, 16], and it was shown that the Iberian lineage is characterized by the presence of a large chromosomal inversion [11]. Whole genome sequencing (WGS) has opened new insights into brucellae taxonomy and phylogeny. The genomic era allowed developing a plethora of new high-resolution methods for the detection and typing of pathogens, and opened new avenues for understanding the full ecological diversity of the genus *Brucella.* Over the last decade, six new species were included in the genus [17]. Further, “atypical” *Brucella* strains were isolated from diverse animal sources, including cold-blooded hosts, such as frogs and fish [18–20] and are likely going to be proposed as new species in the future.

In this study, we performed a comparative analysis of the genomes of *B. suis* biovar 2 with other *Brucella* species to disclose genomic and structural differences between Iberian and Central-European clones. This will allow the assessment of potential factors favoring evolution towards host specialization and contribute to a better understanding of the mechanisms underlying evolution and specialization of Iberian lineages.

## Methods

### Bacterial strains and genetic characterization

A total of 190 *Brucella* spp. strains were used for PCR validation assays, including 22 reference strains representative of eight *Brucella* species and 168 *B. suis* isolates. Eleven of these isolates belonged to biovar 1, 152 to biovar 2, one to biovar 3 and four to biovar 4. Among the *B. suis* biovar 2 isolates, 104 were representative of the Iberian clonal lineage and 48 of the Central-European clonal lineage. Phenotypic characterization of all *Brucella* spp. isolates was performed as previously described [21]. All *B. suis* isolates were previously subjected to PCR-RFLP analysis of the *omp2a* and *omp2b* [22] and *omp31* [23] genes, to assess the different haplotypes, and to target-PCR to confirm the presence of the large inversion in *B. suis* biovar 2 Iberian clonal lineage [11]. All data regarding strain characterization are presented in Additional file 1: Table S1.

### Comparative genomic analysis

The analysis involved 25 brucellae complete genomes (Additional file 2: Table S2), including the five recently sequenced *B.* suis biovar 2 isolates (strains PT09143, PT09172, Bs143CITA, Bs364CITA and Bs396CITA - for details on sequencing, assembly and annotation see Additional file 3 and Additional file 4, Tables S3 and S4).

Multiple sequence alignment of concatenated chromosomes I and II was performed by a superstretch approach using as reference the annotated sequence of strain *B. suis* ATCC 23445: DNA seed 10 matches in windows size of 25 bases, minimal stretch length 60 bases, minimal cut-off for stretch identity of 60% in screening windows of 30 bases was used. The mutation search was performed into the multiple sequence alignment, and included single nucleotide polymorphisms (SNPs; including silent, missense and intergenic mutations) and insertions/deletions (INDELs) variants. To avoid false positives on mutation calling, optimized alignment overlapping stretches settings were used. All query sequences were screened against the reference sequence. A window size of 10 bp was used for determination of pairwise distance, clustering and quality scores for each mutation. The cut-off value for mutation calling on each alignment was set to the 90th percentile of the Poison distribution for score values obtained to each type of mutation. Multiple sequence alignment and mutation analysis were conducted in Kodon V3.62 (Applied Maths, Belgium).

Functional enrichment analysis for missense SNPs among *B. suis* clonal linages was performed on the consolidated semantic annotation obtained from Gene Ontology (GO) [24], the integrative protein signature database (InterPro) [25], and Kyoto Encyclopedia of Genes and Genomes (KEGG) [26] databases, using Blast2GO pipeline (version 2.7.1) [27].

### Evolutionary whole-genome-based studies

To understand the impact of genomic structural rearrangements in the brucellae phylogeny, the relationships among the 25 *Brucella* spp. genomes (Additional file 2: Table S2) were determined using WG-MSA. Clustering analysis was performed using Unweighted Pair Group Method using Arithmetic averages (UPGMA). All positions containing gaps and missing data were eliminated. Chromosomal alignment and clustering analysis were conducted in Kodon V3.62.

The evolutionary history was inferred from genome-wide SNPs (WG-SNP) using Neighbour-Joining (NJ) and Maximum Likelihood (ML). The analysis included the 23 genomes of the eight accepted species and the genome of *Brucella* sp. strain 09RB8910 was used as outgroup (Additional file 2: Table S2). The initial trees were drawn to scale, with branch lengths in the same units as those of the evolutionary distances used to infer the phylogenetic trees. All positions containing gaps and missing data were eliminated. Final phylogenetic trees were built using ML method based on the Tamura-Nei model. The evolutionary analyses were conducted in MEGA6 software package [28].

Evolutionary relationships were also inferred from INDELs information obtained from WG-MSA and coded as binary characters (1 if gap present, 0 if absent). Each instance in the 0/1 matrix corresponds to a single INDEL character, which may reflect either an insertion or a deletion relatively to reference sequence. The quality score was optimized based on a set of parameters to discriminate effective INDELS: quality score, INDEL size, number of neighboring SNPs and INDELs, position of the second different base pair, and INDELs distance to the edge on assembled contig. Likewise, only INDELs with quality scores <1 and sizes bigger than 15 bp were considered. The complete set of the 25 brucellae genomes were included in the analysis (Additional file 2: Table S2). A minimum spanning tree (MST) was generated from a subset of random INDELs using BioNumerics version 6.6 (Applied Maths, Belgium).

### PCR assessment of INDEL events

All the INDELs found to be discriminative of *B. suis* biovar 2 Iberian clonal lineage were assessed by PCR in the collection of the previously described 190 *Brucella* strains. Primers for each lNDEL were designed using the nucleotide positions relative to the reference strain ATCC 23445 and specificity of all the primers was confirmed by BLAST analysis against the published genome sequence of the reference strain. The primer sequences and expected amplicon sizes are provided in Table 1. Genomic DNA from each strain was prepared using the High Pure PCR Template Preparation Kit (Roche Diagnostics, Mannheim, Germany) according to the manufacturer. PCR amplification was performed in a total volume of 25 μl containing 25 ng of DNA, 10× PCR Reaction Buffer, 1 U of Taq DNA polymerase (Promega, USA), 200 mM of each dNTPs and 0.3 mM of each flanking primers. Amplifications were performed in a MyCycler thermal Cycler (Bio-Rad, France). An initial denaturation step at 95 °C for 3 min was followed by 30 cycles of denaturation at 95 °C for 20 s, primer annealing at 56 °C for 30 s and elongation at 72 °C for 30 s. The final extension step was performed at 72 °C for 5 min. Five microliter of amplification products were loaded on a 2% standard agarose gel and run under a voltage of 8 V/cm for 60–90 min. A 100-bp ladder (Invitrogen, USA) was used as molecular size marker.

**Table 1:** List of the primers used for assessment of the INDEL events differentiating the two *B. suis* biovar 2 clonal lineages

INDEL	Primer sequence 5′ → 3’		Chr	Amplified region in ATCC 23445	Affected CDS in ATCC 23445	PCR product expected size (bp)^a^	
						CECL	ICL
SI79506	F R	tgcacacgtagggtcgata cacccagatgttcggctat	I	79,420..80357	BSUIS_A0075	938	260
SI1356057	F R	taggctgcctggaattcatc cggcaagttcacctctgact	I	1,355,436..1356288	Intergenic	853	765
SI1423448	F R	tttcttattccacccgatcc caaaatgttactgcgtgaagc	I	1,423,358..1423655	Intergenic	298	298 (218)
SI1627485	F R	caatccacaggagatcggt gcttcacgggtatcatgtaca	I	1,627,421.. 1,627,600	BSUIS_A1714	180	129
SI2041144	F R	tcggaaatggacgaatatca ttcttgtcgtcggaaatgtc	II	107,306..107533	Intergenic	228	228 (171)
SI2603410	F R	agtggattttggtgcgtttc aagatgagcgggaaatgttg	II	679,423..680774	BSUIS_B0700; BSUIS_B0701	1352	508
LI3234619	F R	tattattcactttgagcggca aactgcaaaagcttggctg	II	1,310,801..1312970	BSUIS_B1354-BSUIS_B1357	2170	174

*CECL* Central-European clonal lineage

*Chr* chromosome

*ICL* Iberian clonal lineage

^a^Values in parenthesis only refer to haplotype 2e strains

## Results and discussion

### Phylogenomic relationships of *B. suis* biovar 2

To understand the genomic specialization observed in the Iberian Peninsula, the genetic structure and evolutionary relationships of the *B. suis* strains isolated in the Iberian Peninsula were assessed by three Whole-Genome based phylogenetic approaches: Multiple Sequence Alignment (WG-MSA); Single Nucleotide Polymorphism distribution (WG-SNP) and insertion and deletion events distribution (WG-INDEL). The phylogenetic analyses involved the full sequence of the five recently published genomes of *B. suis* biovar 2 isolates, and 20 brucellae complete genomes available at the time of the analysis. All genomes with the exception of *B. suis* 686 (biovar 3) have two circular chromosomes (Additional file 2: Table S2).

The WG-MSA analysis was performed for the concatenated chromosomes of each genome and the resultant similarity matrix was used for clustering of the 25 genomes in order to assess the effect of the genomic structural organization on brucellae phylogeny. *Brucella* spp. genomes were grouped into two highly distinct clusters (Additional file 5: Figure S1). It is generally accepted that chromosomal inversions or translocations events may affect the identity values even though little impact is observed in gene content [29]. Indeed, *B. abortus* presents an inversion of 640 kb in chr II [2, 30, 31] and *B. suis* biovar 2 strains PT09172, PT09143 and Bs143CITA present an inversion in chr I with approximately 944 kb [11], as well as the known 210 kb segment of chr I translocated to chr II [1, 2, 11]. Moreover, strain 686 from *B. suis* biovar 3, the unique reference strain with a single chromosome of 3.3 Mbp [32], also revealed an inversion of 790 kb, located between nucleotide #285,192 and #1,074,164 (ATCC 23445 reference positions), in a similar region as the inversion occurring in biovar 2 strains from the Iberian clonal lineage. To the best of our knowledge, this is the first description of a genomic inversion in *B. suis* biovar 3 and of chromosomal structural rearrangements as evolutive features shared by *B. suis* biovar 2 and biovar 3 strains.

**Fig. 1:**
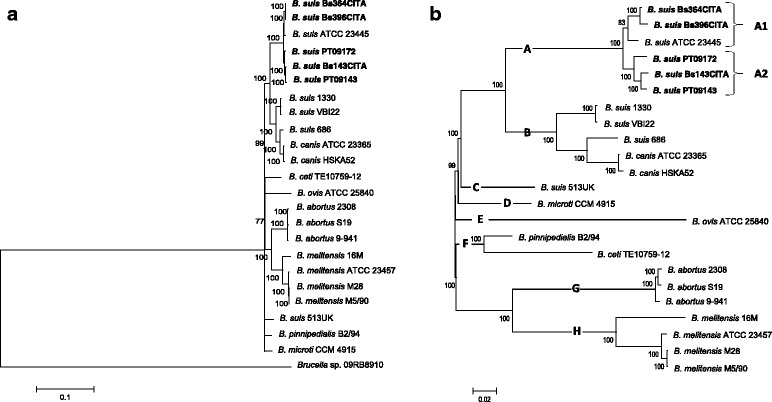
Evolutionary relationships inside genus *Brucella* inferred from WG-SNP analysis. **a** Rooted phylogenetic tree with *Brucella* sp. 09RB8910 as outgroup. The evolutionary history was inferred by using the Maximum Likelihood method based on the Tamura-Nei model [1]. The tree with the highest log likelihood (−581,757.1726) is shown. The percentage of trees in which the associated taxa clustered together is shown next to the branches. Initial tree(s) for the heuristic search were obtained automatically by applying Neighbor-Join and BioNJ algorithms to a matrix of pairwise distances estimated using the Maximum Composite Likelihood (MCL) approach, and then selecting the topology with superior log likelihood value. The tree is drawn to scale, with branch lengths measured in the number of substitutions per site. The analysis involved 25 nucleotide sequences. There were a total of 114,041 positions in the final dataset. **b** Unrooted phylogenetic tree showing in more detail the 23 core *Brucella* genomes, involving a total of 30,255 SNPs in the final dataset and depicting eight clades (A to H). The evolutionary history was inferred by using the Maximum Likelihood method. The tree with the highest log likelihood (−193,253.4180) is shown. The percentage of trees in which the associated taxa clustered together is shown next to the branches. Initial tree(s) for the heuristic search were obtained automatically by applying Neighbor-Join and BioNJ algorithms to a matrix of pairwise distances estimated using the Maximum Composite Likelihood (MCL) approach, and then selecting the topology with superior log likelihood value. The tree is drawn to scale, with branch lengths measured in the number of substitutions per site. Evolutionary analyses were conducted in MEGA6

To unveil the evolutionary history of *B. suis* biovar 2 imprinted on the genomic background, WG-SNP analysis was performed to remove the effect of chromosomal rearrangements (translocations and inversions) on *B. suis* phylogeny*.* The evolutionary history was inferred from a total of 114,031 putative SNPs shared among the 23 more closely related genomes and the genome of the amphibian isolate 09RB8910 (Fig. 1-a; Additional file 6: Table S5). To get a more detailed view on *B. suis* phylogeny, a second tree was built only considering the 30,255 putative SNPs shared among the more closely related *Brucella* species (Fig. 1-b). The evolutionary distances were computed assuming equality of substitution pattern among lineages and of substitution rates among sites using the Maximum Composite Likelihood method and clustered by Neighbor-Joining method (Fig. 1). The unrooted tree sorts the brucellae genomes into eight clades, as follows: the *B. suis* biovar 2 clade (A); the *B. suis-B. canis* clade (B); the *B. suis* biovar 5 clade (C); the *B. microti* clade (D); the *B. ovis* clade (E); the *B. pinnipedialis*-*B. ceti* clade (F); the *B. abortus* clade (G), and the *B. melitensis* clade (H) (Fig. 1-b). These results are in accordance with several phylogenetics studies using WG-SNP or MLST data [2, 3, 33–35]. The *B. suis* biovar 2 isolates formed two distinct phylogenetic groups in clade A, one corresponding to the Central-European clonal lineage (subclade A1) and the other to the Iberian clonal lineage (subclade A2), thus giving additional phylogenetic support at genome level for the occurrence of these clonal lineages already revealed by chromosome I optical maps [11]. Moreover, the evolutionary history inferred from the 944 kb inversion region distinctive of subclade A2 and from 210 kb translocated region of clade A showed no differences in comparison to whole-genome analysis. Alike topologies were observed for coding and non-coding regions (data not shown). Thus, the mechanisms for genomic specialization on the Iberian lineages seem to be independent of the chromosomal rearrangement events observed in these clades.

**Fig. 2:**
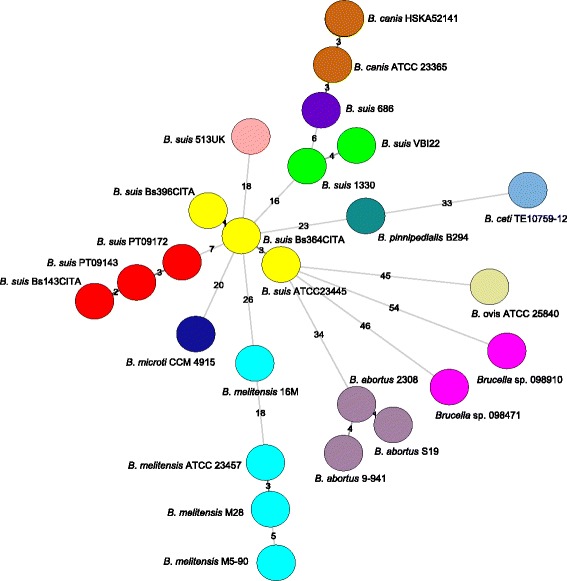
Minimum spanning tree depicting the genomic relationships of *Brucella* species and based on WG-INDELs analysis. A data set of 255 representative INDELs was used, including 7 Iberian ecovar specific indels and a subset of 248 randomly chosen INDELs from the 1131 indels obtained from the comparative analysis of the 25 brucellae genomes. Colour codes are associated with *Brucella* spp. groups and the number of changes between patterns is presented. The MST was constructed with a categorical coefficient using BioNumerics version 6.6

The WG-SNP approach relies on reference-based mapping and does not detect variations in regions that are not present in the reference sequence (i.e. ATCC 23445), and is therefore only suitable for comparison of the core genome shared among all strains. WG-INDELs are largely ignored in phylogenetic reconstruction but provide a suite of markers complementary to nucleotide substitutions with enormous potential for molecular phylogenetics [36–38]. A total of 1, 131 INDELs were revealed in the analysis (Additional file 6: Table S6). In order to confirm the phylogenetic relationships suggested by the former approaches, a minimum spanning tree (MST) was generated from a data set of 255 representative INDELs, which included seven Iberian specific INDELs and a subset of 248 randomly chosen from the 1131 INDELs. Evolutionary relationships based on WG-INDELs are congruent with those from WG-SNP analysis, further revealing *B. suis* biovar 2 Central-European clonal lineage genomes allocated in a well-defined cluster, from which the other *B.suis* biovars and *Brucella* species seem to evolve (Fig. 2). Therefore, we can speculate that biovar 2 Iberian clonal lineage evolved from the Central-European clonal lineage, representing an on-going allopatric speciation process as described for other specialized pathogenic bacteria [38–40]. Since the Iberian clonal lineage is only found in Portugal and Spain south of the Ebro river and the Central-European clonal lineage is only found above this geographical barrier (northeast Spain and other European countries), an ecological differentiation seems to have occurred resulting in the establishment of a new ecovar.

### Comparative genomics of *B. suis* biovar 2

High homology (>98%) between *Brucella* spp. are normally found in association with their preferred hosts and has apparently resulted in adaptive changes over time [41]. Phylogenomic relationships have supported the existence of two *B. suis* biovar 2 phylogenetic groups, which are well separated from *B. suis* biovar 1 and 3. Therefore, the comparative genomics analysis was focused on the detection of distinctive genetic events between those two lineages in comparison with the other *Brucella* species and strains. The inter- and intraspecies comparative analysis revealed several biovar-, haplotype- and strain-specific genetic polymorphisms that can implicate further genetic determinants related to host specificity and genomic specialization in *B. suis*. A set of chromosomal rearrangements and polymorphisms, including SNPs and INDELs, were found and will be further discussed in the following sections.

### Large chromosomal rearrangements in *B. suis* biovar 2 genomes

The chromosomal organization of the six *B. suis* biovar 2 strains was examined by *BamHI* optical mapping and the pairwise alignment between optical and in silico maps allowed the identification of two major chromosomal rearrangements (one translocation event and one inversion) occurring in *B. suis* biovar 2 genomes, pointing out the degree of genome plasticity in *B. suis* species [11].

**Fig. 3:**
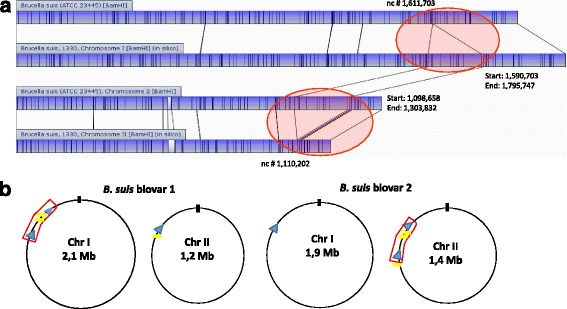
Translocation event in *B. suis* biovar 2 strains. **a** Pairwise alignment of chromosome I and II optical and in silico maps for *B. suis* biovar 2 strain ATCC 23445 and *B. suis* biovar 1 strain 1330. Lines connecting two chromosomal maps indicate discontinuity in the alignment of fragments. The translocated region is highlighted in the red circles; unaligned restriction fragments, representing differences between two chromosomes, are showed in white; blue indicates aligned restriction fragments. **b** Schematic representation of both circular chromosomes. Open red box indicates the translocated region in chromosome I of *B. suis* biovar 1 and in chromosome II of *B. suis* biovar 2; blue triangles symbolize the *rrn* loci; yellow boxes represents the insertion sequence *IS711*

The 210 kb region translocated from Chr I to Chr II, associated to *IS*711, in the six biovar 2 studied genomes (Fig. 3) was firstly described in *B. suis* biovar 2 and 4 reference strains [32]. The translocated region shares 99% similarity with *B.suis* 1330 and encompasses genes with most functions assigned to processes such as transcription, replication and repair, carbohydrate metabolism and metabolism of co-factors and vitamins. Moreover, five tRNAs genes and six ribosomal proteins (L21, L27, L31, L32, L36 and S16) were moved to Chr II, and 14 coding regions in *B. suis* biovar 2 do not have orthologs in *B. suis* 1330 genome.

The most surprising rearrangement was the 944 kb chromosomal inversion present in strains PT09143, PT09172 and Bs143CITA (*B. suis* biovar 2 clade, subclade A1, Iberian clonal lineage) and covering 49% of Chr I [11]. Chr I encodes the majority of the core metabolic machinery for processes such as transcription, translation, and protein synthesis [42]. Although changing gene location, the majority (>95%) of annotated coding regions were found to share 98–100% sequence identity with ATCC 23445 and both genomes from the Central-European clonal lineage.

**Fig. 4:**
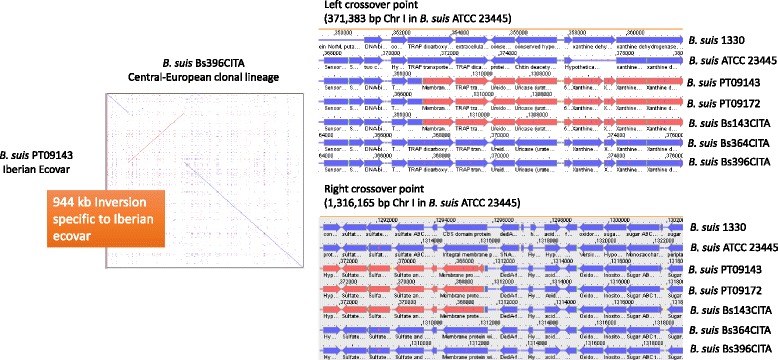
Left and right crossover points of the large inversion present in *B. suis* biovar 2 Iberian ecovar. Dot-blot graphic demonstrating the presence of the 944 kbp inversion in Chr I of *B. suis* biovar 2 strains from Iberian ecovar (1.9 Mbp, aprox. 49%). As an example, the comparison of *B. suis* PT09143 (as the representative strain from Iberian ecovar) with *B. suis* Bs396CITA (representative of the Central-European clonal lineage) is shown. The right and left crossover points are shown. The inversion disrupted a Tripartite ATP-independent periplasmic (TRAP) dicarboxylate transporter, DctM subunit (nucleotide #371,383 in ATCC 23445), and an integral membrane protein TerC (nucleotide #1,316,165 in ATCC 23445), respectively. Chromosomal alignment was conducted in Kodon V3.62

At left and right crossover points, the inversion disrupted a Tripartite ATP-independent periplasmic (TRAP) dicarboxylate transporter, DctM subunit (C-terminus truncated, ortholog to ATCC 23445 BSUIS_A0375 and 1330 BR0344; nucleotide #371383 in ATCC 23445), and an integral membrane protein TerC (N-terminus truncated, ortholog to ATCC 23445 BSUIS_A1382 and 1330 BR1332; nucleotide #1316165 in ATCC 23445), respectively (Fig. 4). The TRAP transporters are a large family of substrate-binding protein (SBP)-dependent secondary transporters found in bacteria and archaea. These transporters have three domains that were defined on the basis of orthology to the three proteins that constitute the Dct system in *Rhodobacter capsulatus* [43], comprising an SBP of the DctP or TAXI families and two integral membrane proteins that form the DctQ and DctM protein families. Orthologs of the three genes can be found in all *Brucella* species (BSUIS_A0374 to BSUIS_A0376 orthologs in ATCC 23445) although *dctQ* and *dctP* were found to be variable within *B. abortus*, *B. canis*, and *B. melitensis*. The gene *dctM* is well conserved in *Brucella* spp. except in *B. suis* biovar 2 strains from Iberian ecovar, probably resulting in the inactivation of this gene in those strains. In *B. melitensis* 16 M the *Dct* operon (BMEI1579- BMEI11581) was predicted to be involved in the transport of mannitol although no experimental evidences exist, but in *R. capsulatus*, *dctP, dctQ,* and *dctM* genes were shown to be essential for C4-dicarboxylate transport [43]. Mannose is both an important precursor in the O-antigen biosynthetic pathway and in the production of the inner core moiety of lipopolysaccharide (LPS) [44, 45]. Loss of the ability to uptake mannitol can influence LPS structure and subsequently host immune responses. At the right crossover, the interrupted ORF codes for TerC, a protein possibly involved in tellurium resistance (inorganic ion transport and metabolism). This membrane protein harbors a CBS domain that is usually associated to enzymatic domains, membrane transporters or DNA-binding domains, playing an important role in host interactions.

### Distinctive SNPs of *B. suis* biovar 2 Iberian ecovar and functional assessment

**Fig. 5:**
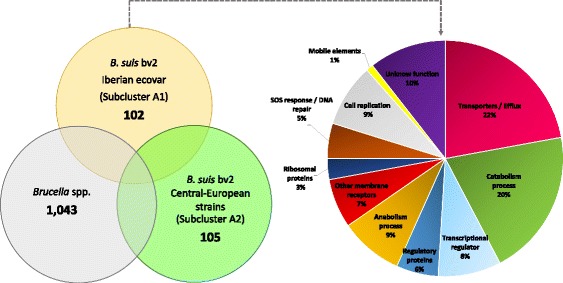
Distribution of putative missense mutations between the two distinct *B. suis* biovar 2 ecovars. The Venn diagram shows the number of unique SNPs between *B. suis* biovar 2 Iberian ecovar (PT09143, PT09172 and Bs143CITA), *B. suis* biovar 2 Central-European lineage (Bs364CITA and Bs396CITA) and the remaining *Brucella* spp. genomes. The associated pie chart shows the breakdown of the functional categories assigned to unique SNPs of Iberian ecovar

The number of SNPs, including intergenic, missense and silent, obtained amongst the brucellae genomes relatively to *B. suis* ATCC 23445 are shown in Table 2 and in Additional file 6: Table S5. Eighty-two percent of the SNPs were identified in coding regions and an average of 51% correspond to missense mutations, affecting genes associated with different classes of cellular functions. The distribution of SNPs along the genome (SNPs per 200 kb) showed no evident differences for the aforementioned chromosomal inversion at Chr I from Iberian *B. suis* biovar 2 strains (Additional file 7: Figure S2). The five *B. suis* biovar 2 strains shared 4087 SNPs. Two-hundred SNPs were unique to strains belonging to the Iberian ecovar (PT09143, PT09172 and Bs143CITA) and 228 discriminate the Central-European strains Bs364CITA and Bs396CITA. Among these, 102 missense SNPs were found exclusively in strains from Iberian ecovar. The three strains of the Iberian ecovar showed a preferential functional enrichment of missense SNPs among the annotated genes associated to transporter/ efflux systems, other membrane receptors, metabolism processes, transcriptional regulators, regulatory proteins, cell replication, SOS response/DNA repair and ribosomal proteins (Fig. 5). The majority of discriminative mutations was associated to membrane related molecules (29%; *n* = 30) and enzymes involved in catabolism process (20%; *n* = 21), which may be related to molecular tropism to a specific environment or animal host.

**Table 2:** Resume of mutation analysis of brucellae genomes using *B.suis* ATCC 23445 as reference

Strain Identification	Biovar	Host	INDELS	Single Nucleotide Polymorphisms			
				Intergenic	Missense	Silent	Total
*B. suis* ATCC 23445	2	Hare	**–**	**–**	**–**	**–**	–
*B. suis* Bs364CITA	2	Wild boar	11	165	256	146	567
*B. suis* Bs396CITA	2	Wild boar	13	255	287	161	703
*B. suis* PT09172	2	Wild boar	18	225	420	260	905
*B. suis* PT09143	2	Wild boar	19	281	395	239	915
*B. suis* Bs143CITA	2	Wild boar	20	295	434	268	997
*B. suis* VBI22	1	Swine	77	714	2687	1523	4924
*B. suis* 1330	1	Swine	78	729	2639	1557	4925
*B. pinnipedialis* B2/94	not Applied	Dolphin	100	763	2911	1517	5191
*B. suis* 686	3	Seal	77	764	2951	1661	5376
*B. canis* HSKA52141	not applied	Dog	72	799	2985	1632	5416
*B. microti* CCM 4915	not applied	Dog	80	985	2817	1644	5446
*B. suis* 513UK	not applied	Wild rodent	75	840	3025	1596	5461
*B. canis* ATCC 23365	5	Wild rodent	71	826	2956	1706	5488
*B. ceti* TE10759–12	not applied	Seal	171	1002	3740	2017	6759
*B. abortus* 2308	1	Cattle	119	1152	4452	2465	8069
*B. abortus* 9–941	1	Cattle	121	1151	4465	2454	8070
*B. abortus* S19	1	Vaccine	118	1136	4478	2465	8079
*B. melitensis* ATCC 23457	2	Goat	104	1196	4662	2563	8421
*B. melitensis* M28	not applied	Sheep	104	1199	4687	2567	8453
*B. ovis* ATCC 25840	1	Sheep	225	1227	4529	2714	8470
*B. melitensis* M5–90	1	Sheep	108	1204	4707	2601	8512
*B. melitensis* 16 M	1	Goat	115	1117	5594	1994	8705
*Brucella* sp. 09RB8910	not applied	African bullfrog	207	6275	13,528	37,119	56,922
*Brucella* sp. 09RB8471	not applied	African bullfrog	260	6525	14,554	37,589	58,668

### INDELs differentiative of biovar 2 Iberian ecovar

The comparative analysis of the effective INDELS disclosed 10 INDELs in coding regions, including one Large INDEL (LI ≥ 1 kb), and three intergenic INDELs were found to be specific of the Iberian ecovar strains PT09143, PT09172 and Bs143CITA (Additional file 6: Table S7). The LI, with 1996 bp in Chr II (LI3234619), is presented in one region encoding ORFs BSUIS_B1354 to BSUIS_B1357 in ATCC 23445 within one *Brucella* flagellar gene cluster. BSUIS_B1354, BSUIS_B1355 and BSUIS_B1357 (BRA1127, BRA1128 and BRA1130 orthologs in 1330) code for hypothetical proteins without any specific functional domains. BSUIS_B1356 (BRA1129 ortholog in 1330) is predicted to code a flagellar protein FlgJ, possessing an N-terminal domain responsible for proper rod assembly [46]. Primers (LI3234619-F and LI3234619-R) directed to flanking regions were used for INDEL search, confirming the presence of this INDEL in strains PT09143, PT09172, Bs143CITA and in the 104 biovar 2 strains from the Iberian ecovar. Although brucellae were considered non-motile bacteria for a long time, it has been reported that *B. melitensis* produces a functional flagellum with the characteristics of a sheathed flagellum-like structure, which is produced only transiently at the end of the exponential phase of growth [47, 48]. Moreover, recently “atypical” *Brucella* sp. strains were isolated from amphibians showing for the first time high motility [18, 49]. The bacterial flagellum is a complex apparatus composed of at least 31 different proteins, which are similarly organized in all brucellae genomes, with genes distributed in three clusters on the small chromosome [18, 35]. However, a recent comparative gene-based analysis revealed that the majority of these flagellar genes were fully functional in all motile *Brucella sp.* strains analyzed, but non-motile brucellae presented a number of pseudogenized genes [18]. The *flgJ* gene seems to be functional in all brucellae except in biovar 2 strains from the Iberian ecovar. It is documented that flagellar genes are required for the establishment of in vivo infection in mice and goats [42, 44]. Consequently, the inactivation (due to point mutations or small indels) or loss of key flagellar genes would influence the formation of a functional flagellum and therefore several functions can be affected, such as protein export or adhesion [50].

Among the nine Small INDELs (SI < 1-kb) in coding regions, three specific SIs were further evaluated due to their location in genome and eventual importance in evolution and genomic specialization of *B. suis* biovar 2 Iberian ecovar. SI79420 (678 bp) occurs within an ORF coding for an outer membrane protein ortholog to ATCC 23445 BSUIS_A0075 and 1330 BR0072, which was described as a putative autotransporter adhesin [35, 42]. In fact, different proteins belonging to the autotransporter family have been identified in *Brucella* genomes, sharing a common domain organization: an N-terminal secretion signal, a divergent and functional domain (passenger domain) and a conserved C-terminal region [35, 51–53]. The alignment of the ortholog genes in the different species indicated that this INDEL occurs within the passenger domain, showing a range in similarity (at the nucleotide level) with BSUIS_A0075, between 98% (Bs364CITA and Bs396CITA), and 72% (PT09143, PT09172 and Bs143CITA). SI79420 probably caused the inactivation of the protein but it remains to be seen if this protein is functional or if differences within the passenger domain contribute to host or tissue specificity or clinical manifestations in wild boars or pigs. SI1627421 (59 bp) affects a permease, ortholog to ATCC 23445 BSUIS_A1714 and 1330 BR1873, associated to autotransporter proteins. The three strains from the Iberian clonal lineage presented the 59 bp INDEL next to the C-terminus. Since almost all of the known autotransporters are involved in functions related with the invasion process, the difference in the number of active autotransporters, and the variation within them, may play a role in the ability of each species to interact with its host and may thus be an important contributor to virulence [35, 42]. Lastly, SI2603410 (844 bp) represent an event that causes the elimination of one insertion sequence. The *IS711* insertion sequence is unique to *Brucella* species and the number of copies in the genome varies between species and biovars. This is regarded as a key determinant in genome plasticity and was suggested to provide significant adaptive changes to genomes. Seven complete copies of this insertion sequence are recognized in *B. suis* 1330 and 13 in ATCC 23445 [34], as well as in strains Bs364CITA and Bs396CITA. However, Iberian ecovar strains PT09143, PT09172 and Bs143CITA present 12 copies of this insertion element, including *orfA* and *orfB* genes. From the three intergenic INDELs, the SI1356057 (88 bp) is located between a GntR family transcriptional regulator and a ketol-acid reductoisomerase. In ATCC 23445, a CDS coding for a hypothetical protein (BSUIS_A1430) is annotated among the GntR family transcriptional regulator (BSUIS_A1429), and the ketol-acid reductoisomerase (BSUIS_A1431). Orthologous of the three genes are also present in both Central–European strains, but BSUIS_A1430 ortholog is missing in PT09143, PT0172 and Bs143CITA. Additionally, two intergenic SIs specific for strains PT09143 and Bs143CITA (Iberian ecovar, haplotype 2e), one in Chr I with 77 bp (SI1423448), and other in Chr II with 47 bp (SI2041144) were found. Nevertheless, no frameshift is expected or no promoter region seemed to be affected by those two described INDELs.

The six abovementioned SIs were searched by targeted-PCRs in the 190 *Brucella* strains and it was confirmed that those events were specific of Iberian ecovar (Additional file 1: Table S1).

Finally, no INDELs were found to affect genes known to participate in virulence, such as lipopolysaccharide biosynthesis, two-component regulatory system BvrR/BvrS, type IV secretion system VirB or erythritol catabolic pathway.

## Conclusion

In this work, a full genome comparative analysis of five *B. suis* biovar 2 strains isolated from wild boars belonging to the main circulating clonal lineages in the Iberian Peninsula and publicly available *Brucella* spp. genomes was performed. *B. suis* biovar 2 strains from the Iberian clonal lineage could be differentiated from strains from the Central-European clonal lineage not only by the presence of one large inversion in Chr I but also by a number of specific SNPs, deletions and insertions. Additionally, the mutational enrichment of the Iberian lineage was to genes encoding membrane proteins with potential of interaction with external stimulus, and to genes with impact on the pathogen metabolism. However, further studies are needs to understand the metabolic consequences of these disarrangements.

In summary, whole-genome analyses support that the *B. suis* biovar 2 Iberian clonal lineage evolved from the Central-European lineage and suggest that genomic specialization of this pathogen in the Iberian Peninsula is independent of a specific genomic event(s), but instead driven by allopatric speciation, resulting in the establishment of an ecovar.

## Additional files

Additional file 1: Table S1. *Brucella suis* population genetic diversity information. (XLSX 29 kb)

Additional file 2: Table S2. List of genomes used for phylogenetic and comparative genomic analysis.) (DOCX 17 kb)

Additional file 3: De novo sequencing and assembly of *B. suis* biovar 2 strains PT09143, PT09172, Bs143CITA, Bs364CITA and Bs396CITA. (DOCX 24 kb)

Additional file 4: Table S3. Summary statistics for assembly of five *B. suis* biovar 2 strains isolates from wild boars; **Table S4**. General features of *B. suis* biovar 2 genomes. (XLSX 13 kb)

Additional file 5: Figure S1. Comparative chromosome mapping of 25 *Brucella* spp. genomes. Genomic alignment of concatenated chromosomes I and II was performed by superstretch approach: DNA seed 10 matches in windows size of 25 bases, minimal stretch length 60 bases, minimal cut-off for stretch identity of 60% in screening windows of 30 bases was used. Each cell in the matrix displays the identity score, with a corresponding color scale. The left-to-right diagonal of the matrix contains those cells representing the comparison of sequences compared to themselves. The value in each cell represent the percentage of repetitive regions for that sequence. The scale goes from black, corresponding with 100% identity, over blue towards white (0% identity). Clustering analysis using UPGMA. All positions containing gaps and missing data were eliminated. (PDF 43 kb)

Additional file 6: Table S5. List of SNPs disclosed in the comparative genomic analysis of 25 *Brucella* genomes using as reference the annotated sequence of strain *B. suis* ATCC 23445; **Table S6.** List of INDELs disclosed in the comparative genomic analysis of 25 *Brucella* genomes using as reference the annotated sequence of strain *B. suis* ATCC 23445; **Table S7.** List of INDELs differentiative of *B. suis* biovar 2 Iberian ecovar. (XLSX 37286 kb)

Additional file 7: Figure S2. Distribution of SNPs along the genome (SNPs per 0,2 Mb). (PDF 194 kb)

## Abbreviations

BLAST: basic local alignment search tool
CDS: coding DNA sequence
Chr: chromosome
GO: gene ontology
INDEL: insertions/deletions
InterPro: integrative protein signature database
KEGG: Kyoto encyclopedia of genes and genomes
LPS: lipopolysaccharide
ML: maximum likelihood
MLST: multiple locus sequence typing
MLVA: multiple-locus variable number tandem repeat analysis
MSA: multiple sequence alignment
MST: minimum spanning tree
NCBI: National Center for Biotechnology Information
NJ: neighbour-joining
ORFs: opening reading frames
PCR: polymerase chain reaction
RFLP: restriction fragment length polymorphism
SNP: single nucleotide polymorphism
UPGMA: unweighted pair group method using arithmetic averages
WG: whole genome
