# Supplementary material for: Evolution and genome specialization of Brucella suis biovar 2 Iberian lineages
Source: BMC Genomics. 2017 Sep 12;18:726. doi: 10.1186/s12864-017-4113-8 (PMC5596481; doi:10.1186/s12864-017-4113-8)
Supplement: Supplementary file 2 — List of genomes used for phylogenetic and comparative genomic analysis.) (DOCX 17 kb) [file 12864_2017_4113_MOESM2_ESM.docx]

**Additional file 2**

**Table S2**

List of genomes used for phylogenetic and comparative genomic analysis

| Genome | Biovar | Host | NCBI Reference sequence  (Chr I/ Chr II) |
| --- | --- | --- | --- |
| *B. suis* 1330 | 1 | Swine | NC_004310.3/ NC_004311.2 |
| *B. suis* VBI22 | 1 | Swine | NC_016797.1/ NC_016775.1 |
| *B. suis* ATCC 23445 | 2 | Hare | NC_010169.1/ NC_010167.1 |
| *B. suis* Bs364CITA | 2 | Wild boar | NZ_CP007697.1/ NZ_CP007698.1 |
| *B. suis* Bs396CITA | 2 | Wild boar | NZ_CP007720.1/ NZ_CP007721.1 |
| *B. suis* PT09172 | 2 | Wild boar | NZ_CP007693.1/ NZ_CP007694.1 |
| *B. suis* PT09143 | 2 | Wild boar | NZ_CP007691.1/ NZ_CP007692.1 |
| *B. suis* Bs143CITA | 2 | Wild boar | NZ_CP007695.1/ NZ_CP007696.1 |
| *B. suis* bv.3 str. 686 | 3 | Seal | NZ_CP007719.1/ NZ_CP007718.1 |
| *B. suis* 513UK | 5 | Wild boar | NZ_CP007717.1/ NZ_CP007716.1 |
| *B. abortus* 2308 | 1 | Cattle | NC_007618.1/ NC_007624.1 |
| *B. abortus* 9-941 | 1 | Cattle | NC_006932.1/ NC_006933.1 |
| *B. abortus* S19 | 1 | Vaccine | NC_010742.1/ NC_010740.1 |
| *B. melitensis* 16M | 1 | Goat | NC_003317.1/ NC_003318.1 |
| *B. melitensis* M5-90 | 1 | Sheep | NC_017246.1/ NC_017246.1 |
| *B. melitensis* M28 | 1 | Sheep | NC_017244.1/ NC_017244.1 |
| *B. melitensis* ATCC 23457 | 2 | Goat | NC_012441.1/ NC_012442.1 |
| *B. canis* HSKA52141 | Not applicable | Dog | NC_016778.1/ NC_016796.1 |
| *B. canis* ATCC 23365 | Not applicable | Dog | NC_010103.1/ NC_010104.1 |
| *B. ceti* TE10759-12 | Not applicable | Seal | NC_022905.1/ NC_022906.1 |
| *B. pinnipedialis* B2/94 | Not applicable | Dolphin | NC_015857.1/ NC_015858.1 |
| *B. microti* CCM 4915 | Not applicable | Wild rodent | NC_013119.1/ NC_013118.1 |
| *B. ovis* ATCC 25840 | Not applicable | Sheep | NC_009505.1/ NC_009504.1 |
| *Brucella* sp. 09RB8471 | Not applicable | Africa bullfrog | NZ_CP019346.1/ NZ_CP019347.1 |
| *Brucella* sp. 09RB8910 | Not applicable | Africa bullfrog | NZ_CP019390.1/ NZ_CP019391.1 |
