# Supplementary material for: Evolution and genome specialization of Brucella suis biovar 2 Iberian lineages
Source: BMC Genomics. 2017 Sep 12;18:726. doi: 10.1186/s12864-017-4113-8 (PMC5596481; doi:10.1186/s12864-017-4113-8)
Supplement: Supplementary file 3 — De novo sequencing and assembly of B. suis biovar 2 strains PT09143, PT09172, Bs143CITA, Bs364CITA and Bs396CITA. (DOCX 24 kb) [file 12864_2017_4113_MOESM3_ESM.docx]

**Additional file 3**

***De novo* sequencing, assembly and functional annotation of *B. suis* biovar 2 strains PT09143, PT09172, Bs143CITA, Bs364CITA and Bs396CITA**

Total genomic DNA was extracted and purified using the PureLink Genomic DNA Kit (Invitrogen – Life Technologies, USA). An aliquot of the DNA was subjected for analysis using the Bioanalyzer (Agilent technologies) and was confirmed for no degradation. An aliquot of 10 µg of DNA was used for the sequencing and the remaining stock was maintained for further sequencing and completion of gaps. Genomic libraries were created using Truseq DNA sample preparation kit and genomic sequences obtained by Illumina HiSeq 2000 technology with a paired-end 35-bp protocol. Only reads with Phred score Q > 30 were considered for *de novo* assembly (depth coverage aptx: 100X), using de Bruijn graph method (Velvet version 1.2.09) [1]. Summary of statistics of assembly process is presented in Additional file 3: Table S2. Visual inspection of alignment of fastq reads for each contig was performed using Table 1.14.04.10. Scaffolding was guided by optical mapping method (MapSolver version 3.2; OpGen Technologies, Inc.) and gap filling was performed by PCR and Sanger method [2]. Low coverage regions and all indels (in comparison with *B. suis* ATCC 23445) were confirmed by Sanger resequencing. Chromosome-wide comparisons of those five strains and reference strains *B. suis* ATCC 23445 and 1330 was made using approach based in Mummer algorithm implemented at Kodon V3.62. Genomic alignment of concatenated chromosome was performed by superstretch approach: DNA seed 15 matches in windows size of 25 bases, minimal stretch length 60 bases, and minimal cut-off for stretch identity of 60% in a screening window of 30 bases was used.

Genomic DNA from the five strains, and from the reference strain *B. suis* ATCC 23445 was extracted and optical maps were prepared using the Argus Whole Genome Mapping System (OpGen, Inc., Madison, USA). It was produced a consensus optical map for each strain from the restriction pattern obtained at single-molecule level. An optical map is a linear representation of each chromosome, showing restriction cut sites and fragment sizes. Optical maps analysis were performed using MapSolver version 3.2 (OpGen Technologies, Inc.). DNA sequences of chromosome I and II from ATCC 23445 (CP000911, CP000912), and *B. suis* biovar 1 strain 1330 (CP002997, CP002998) were obtained by FTP from the NCBI genome database, and *in silico BamHI* restriction maps for each chromosome were created using MapSolver. Sequence-to-map comparisons were performed using MapSolver software (OpGen Technologies, Inc.). Sequence FASTA files from each chromosome were converted to in silico restriction maps for direct comparison to the Optical maps. Comparisons were accomplished by aligning the sequence with the Optical maps according to their restriction fragment pattern. Alignments were generated with a dynamic programming algorithm which finds the optimal location, or placement, of a sequence contig by first performing a global alignment of the sequence contig against the Optical map.

The ORF detection and primary functional annotation was made through Rapid Annotations using Subsystems Technology (RAST) [3], 23S rRNA and tRNA genes were identified using RNAmmer [4] and tRNAscan-SE 1.21 [5], respectively (Additional file 4, Table S3). Functional enrichment was performed using Gene Ontology (GO) [6], the integrative protein signature database (InterPro) [7] and Kyoto Encyclopedia of Genes and Genomes (KEGG) [8] databases, using Blast2GO pipeline (version 2.7.1) [9]. Search for phage at the sequenced genomes and strains *B. suis* ATCC 23445 and 1330 was made using PHAST (PHAge Search Tool) [10]. For ORFs without functional information attributed by RAST server nor Blast2GO, annotation was obtained by query with BLASTn algorithm against nr/nt database from NCBI (May 10, 2014) using standard configuration. Functional annotation of ORFs were manually checked for incongruences between Gene Ontology, Enzyme Codes, InterPro and KEGG databases assignments. Data consolidation, mining and querying was performed using MySQL InnoDB engine (5.0.95). Scripting and parsing was made using Python (2.7.3) [11].

**References**

1. Zerbino DR, Birney E. 2008. Velvet: algorithms for de novo short read assembly using de Bruijn graphs. Genome Res. 18:821– 829.
2. Sanger F, Nicklen S, Coulson AR. 1977. DNA sequencing with chain-terminating inhibitors. Proc. Natl. Acad. Sci. U. S. A. 74:5463–5467.
3. Aziz RK, Bartels D, Best AA, DeJongh M, Disz T, Edwards RA, Formsma K, Gerdes S, Glass EM, Kubal M, Meyer F, Olsen GJ, Olson R, Osterman AL, Overbeek RA, McNeil LK, Paarmann D, Paczian T, Parrello B, Pusch GD, Reich C, Stevens R, Vassieva O, Vonstein V, Wilke A, Zagnitko O. The RAST server: Rapid Annotations using Subsystems Technology. BMC Genomics.2008;9:75.
4. Lagesen K, Hallin P, Rødland EA, Staerfeldt HH, Rognes T, Ussery DW. RNAmmer: consistent and rapid annotation of ribosomal RNA genes. Nucleic Acids Res. 2007;5:3100–08.
5. Lowe TM, Eddy SR. tRNAscan-SE: a program for improved detection of transfer RNA genes in genomic sequence. Nucleic Acids Res. 1997;25: 955–64.
6. Ashburner M, Ball CA, Blake JA, Botstein D, Butler H, Cherry JM, Davis AP, Dolinski K, Dwight SS, Eppig JT, Harris MA, Hill DP, Issel-Tarver L, Kasarskis A, Lewis S, Matese JC, Richardson JE, Ringwald M, Rubin GM, Sherlock G. Gene ontology: tool for the unification of biology. Nat Genet. 2000;25(1):25-9. Online at Nature Genetics.
7. Hunter S, Apweiler R, Attwood TK, Bairoch A, Bateman A, Binns D, Bork P, Das U, Daugherty L, Duquenne L, Finn RD, Gough J, Haft D, Hulo N, Kahn D, Kelly E, Laugraud A, Letunic I, Lonsdale D, Lopez R, Madera M, Maslen J, McAnulla C, McDowall J, Mistry J, Mitchell A, Mulder N, Natale D, Orengo C, Quinn AF, Selengut JD, Sigrist CJ, Thimma M, Thomas PD, Valentin F, Wilson D, Wu CH, Yeats C. InterPro: the integrative protein signature database. Nucleic Acids Res. 2009; 37:D211-5. doi: 10.1093/nar/gkn785.
8. Kanehisa M and Goto S. KEGG: Kyoto Encyclopedia of Genes and Genomes. Nucleic Acids Res. 2000; 28(1): 27–30.
9. Götz S, García-Gómez JM, Terol J, Williams TD, Nagaraj SH, Nueda MJ, Robles M, Talón M, Dopazo J, Conesa A. High-throughput functional annotation and data mining with the Blast2GO suite. Nucleic Acids Res. 2008;36(10): 3420-35).
10. Zhou Y, Liang Y, Lynch K, Dennis JJ, Wishart DS. PHAST: A Fast Phage Search Tool” Nucl. Acids Res. 2011;39(suppl 2):347-52
11. Van Rossum, G. 2007. Python programming language. In USENIX Annual Technical Conference.
