# Supplementary figures and images for: Evolution and genome specialization of Brucella suis biovar 2 Iberian lineages
Source: BMC Genomics. 2017 Sep 12;18:726. doi: 10.1186/s12864-017-4113-8 (PMC5596481; doi:10.1186/s12864-017-4113-8)

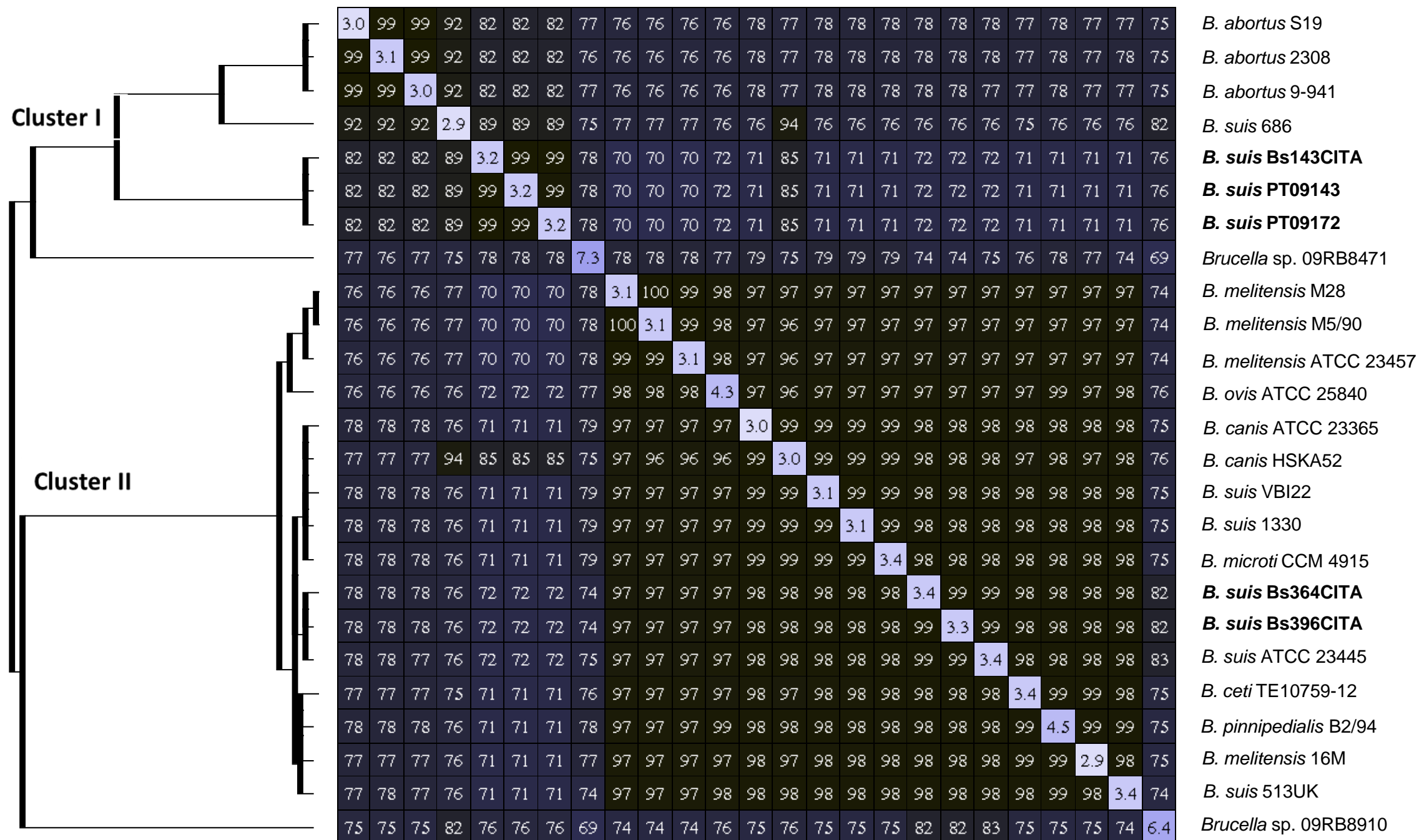

Supplement: Supplementary file 5 — Comparative chromosome mapping of 25 Brucella spp. genomes. Genomic alignment of concatenated chromosomes I and II was performed by superstretch approach: DNA seed 10 matches in windows size of 25 bases, minimal stretch length 60 bases, minimal cut-off for stretch identity of 60% in screening windows of 30 bases was used. Each cell in the matrix displays the identity score, with a corresponding color scale. The left-to-right diagonal of the matrix contains those cells representing the comparison of sequences compared to themselves. The value in each cell represent the percentage of repetitive regions for that sequence. The scale goes from black, corresponding with 100% identity, over blue towards white (0% identity). Clustering analysis using UPGMA. All positions containing gaps and missing data were eliminated. (PDF 43 kb) [file 12864_2017_4113_MOESM5_ESM.pdf]

Additional file 7. Figure S2  
Distribution of SNPs along the genome (SNPs per 0,2 Mbp)

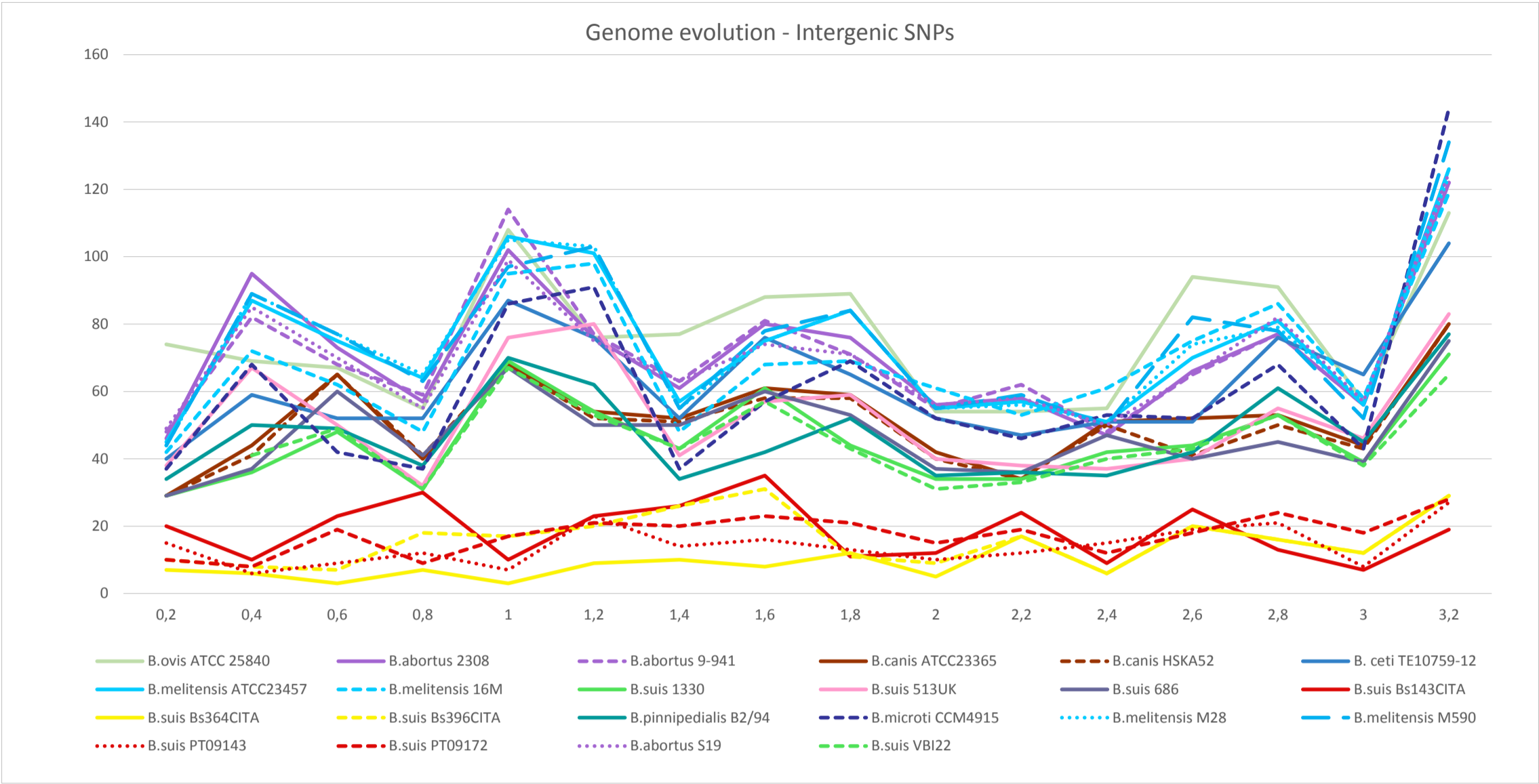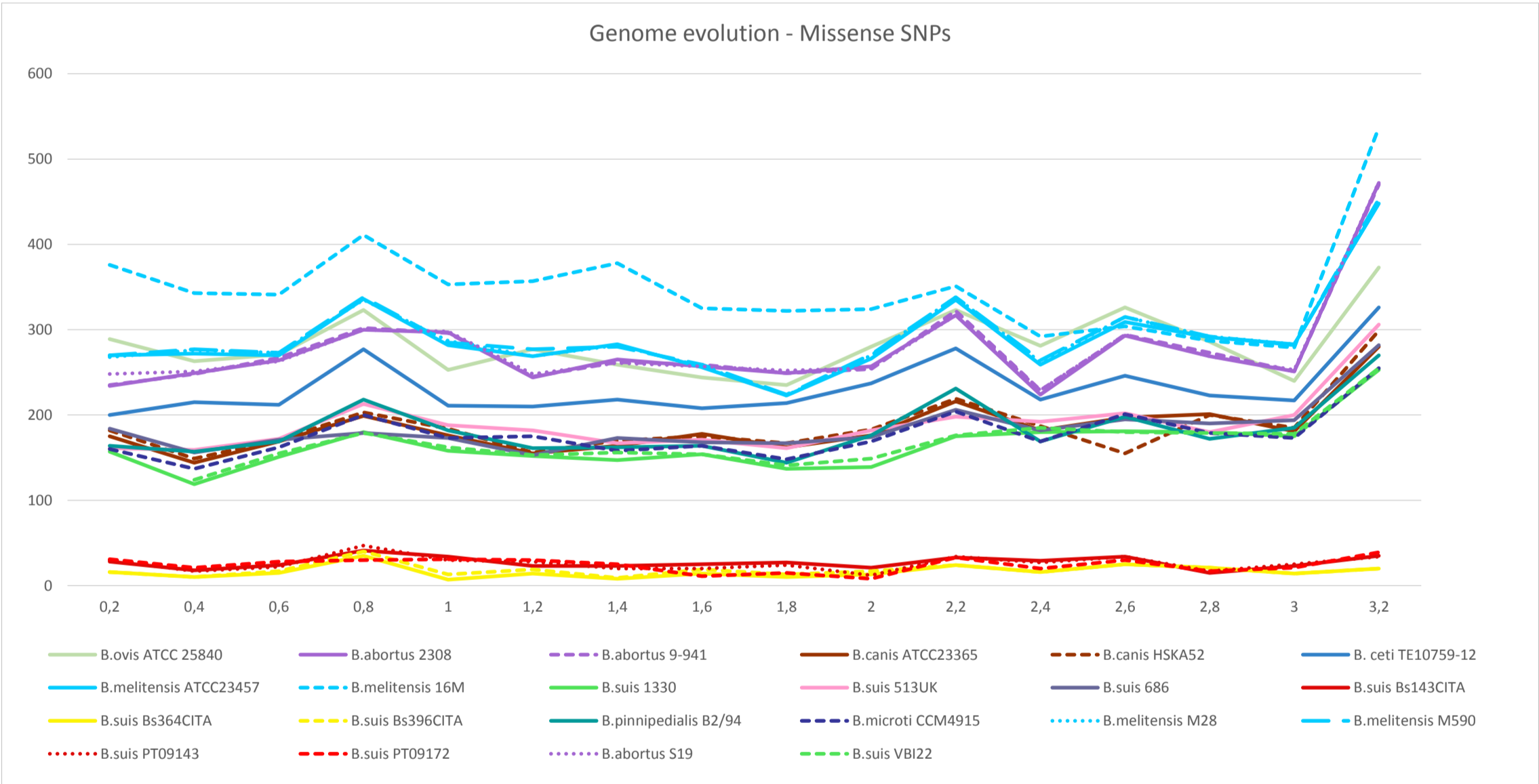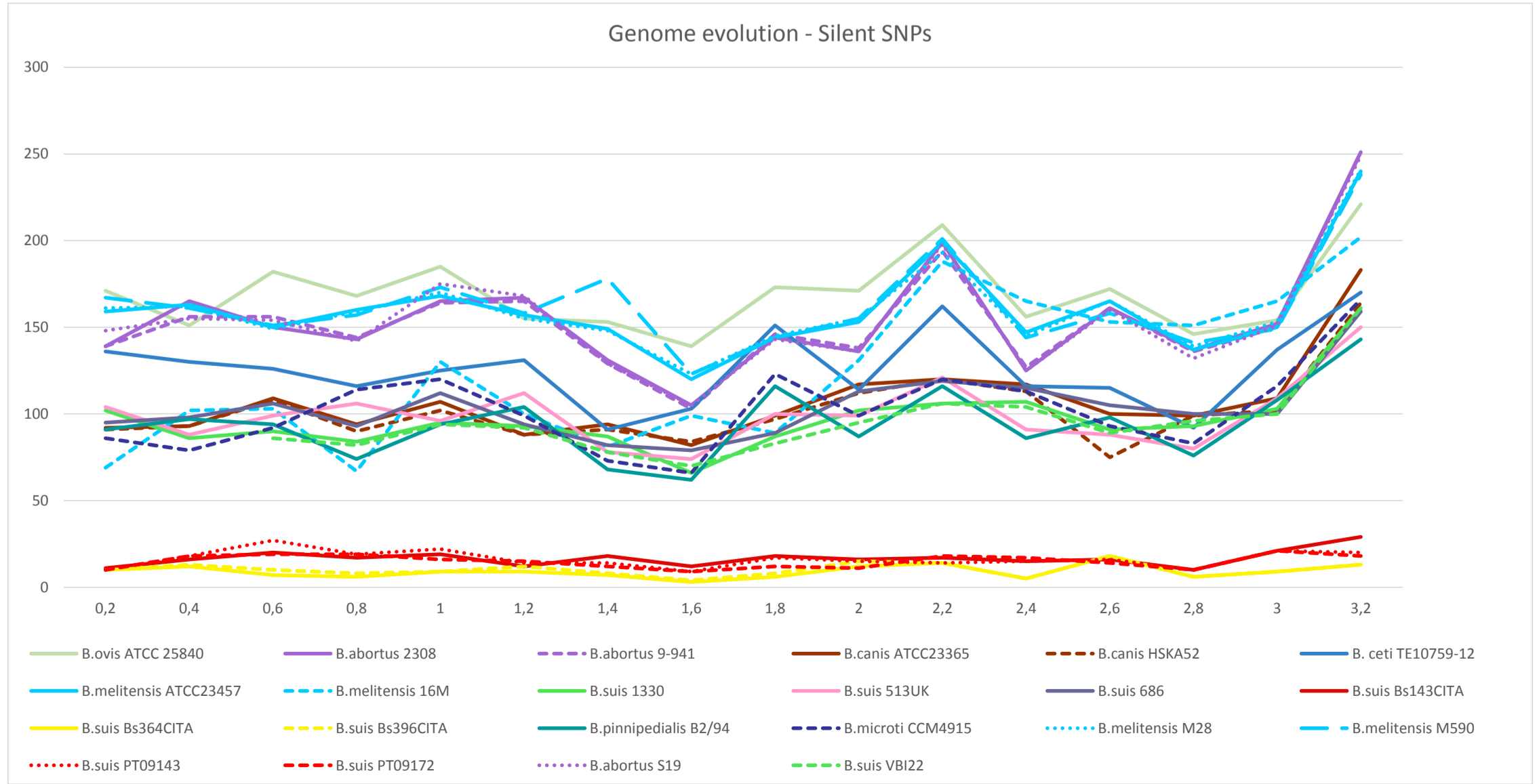

Supplement: Supplementary file 7 — Distribution of SNPs along the genome (SNPs per 0,2 Mb). (PDF 194 kb) [file 12864_2017_4113_MOESM7_ESM.pdf]
